# Abridging CMOS Technology

**DOI:** 10.3390/nano12234245

**Published:** 2022-11-29

**Authors:** Hei Wong

**Affiliations:** Department of Electrical Engineering, City University of Hong Kong, Hong Kong, China; heiwong@ieee.org

Whether from a device physics, fabrication technology, or process economics point of view, the practice of downsizing silicon-based CMOS devices will soon end. The commercial technological node may be decreased to 1 nm from the chip density point of view, but the physical dimension of different linewidths in integrated circuits, such as channel width and channel length, should remain at around 4 nm [1]. New revolutionized materials and technologies for further integrated electronics are on the horizon. However, considering the nanoscale in size, the giga-scale in integration density, the complexity of fabrication technology, as well as the fact that widespread application of the present CMOS technology was resulted from the relentless advancement and innovation of over seven decades, the emerging materials and devices are unlikely to replace CMOS technology in the short term [1,2]. CMOS technology based on two-dimensional (2D) materials will likely not be available for mainstream ICs within this decade. It is possible that the existing CMOS technology will remain the mainstream integration technology for decades to come; newly discovered materials, such as emerging 2D materials in various forms, together with the achievements in atomic-level fabrication technology, will be available as technological options for overcoming some of the constraints in CMOS devices and fabrication technology, and could enrich and enhance certain aspects of CMOS technology.

This Special Issue presents reports on the latest progress on current issues in CMOS device downsizing and prospective views on the more-than-Moore options for future CMOS integration. This first volume (the second issue is in progress) consists of nine papers, including three review papers and six regular papers, from different research teams worldwide. We have contributions addressing topical issues of the CMOS device structure downsizing towards the ultimate scale such as nanosheet transistor design and modeling [3] and variability issues related to the surface roughness of ultrathin films [4]. Nanosheet transistor has been considered as the ultimate device structure at the end of Moore’s law [5], but the calculations made by Wong and Kakushima indicate that the minimum achievable thickness of gate dielectric film could hinder the further downsizing of nanosheet transistor below 5 nm in physical gate length [3]. In the next few technological nodes, we will encounter a scenario in which the gate dielectric thickness and the channel thickness are scaled closer to the interface layer thickness or the surface roughness value of these ultrathin films. The interface and surface roughness should have significantly impact on the device characteristics, process uniformity, and thus result in the yield degradation of the ultra-large-scale integrated circuits. One paper in this Special Issue discusses the impact of the surface roughness of subnanometer EOT high-k gate dielectrics on the electrical characteristic variabilities [4]. The reliability challenges of 7 nm CMOS technology and beyond are also addressed [6,7]. Zhu et al. present an effective scheme for electrostatic discharge protection for 7 nm FinFET and beyond [6]. Before 2D materials can completely replace silicon material in semiconductor device and integrated-circuit fabrication, the CMOS community has a consensus that Moore’s Law in terms of integration density per unit area will continue to advance for several generations with the 3D stacking or heterogeneous integration (HI) technology [1,7]. Besides the benefit of higher integration density, HI technologies enable the integration of different functionality building blocks that can be fabricated using different substrate materials and technologies. This is expected to be the most important strategy in the coming decades for integrating high-performance 2D-material-based devices or modules with the existing silicon-based CMOS technology. Wang’s team provides a comprehensive review of heterogeneous integration (HI) technology and related reliability issues [7]. In particular, they highlight several successful HI devices and technologies, such as vertical magnetic-cored inductors, metal-wall structures based on CMOS back-end-of-line (BEOL) technology, graphene nano-electromechanical system (NEMS) switches, graphene interconnects, and nano-crossbar arrays for electrostatic discharge (ESD) protection.

Three contributions report on non-CMOS-based sensors that have a high potential to be integrated with CMOS technology via HI. Our CMOS ICs will be enriched with 2D-material-based devices, sensors, and transducers to provide better man–environment interaction through HI technology. Moorthy and Srivastava conducted device modeling and parameter optimization for carbon-nanotube-based solar cells and photodiodes [8]. Shi et al. proposed a small-sized film bulk acoustic resonator (FBAR) structure based on 2D photonic crystals [9]. Ding and co-workers developed a simulation framework for nanoscale-phase change memory (PCM) designs [10]. They also address several nanofabrication issues, such as the crystallization and nucleation of nanoscale materials during thin-film growth.

Over the last few decades, the microelectronics industry, driven by the more-than-Moore initiatives, has actively explored the potential of fully functional integration of semiconductor-based devices, beyond digital logic and memory, such as radio-frequency frontends, and analog circuits, biochips, and sensors on the same chip [1,2]. In this aspect, Filipovic and Selberherr [11] provide a comprehensive review of the state-of-the-art gas sensing technologies, including new sensing materials and the techniques for their fabrication, such as semiconductor metal oxide (SMO), graphene oxide, reduced graphene oxide, transition-metal dichalcogenides (TMDs), phosphorene, and MXenes, novel device structures and related gas sensing/detection techniques, and issues regarding the integration of these sensors with CMOS technology.

This Special Issue also includes a review paper by Knobloch, Selberherr, and Grasser entitled “Challenges for Nanoscale CMOS Logic Based on Two-Dimensional Materials” [12]. This paper addresses most of the themes, concerns, and challenges related to integrating CMOS technology with emerging materials and technologies. They highlight the advantages of 2D materials and present a comprehensive review of the recent attempts to fabricate field-effect transistors based on 2D materials. They also critically discuss fabrication issues, such as the growth of high-quality 2D materials, the formation of low-resistive contacts to 2D semiconductors, potential gate stack materials for high channel mobilities, and issues related to wafer-scale process integration. Knobloch and co-workers further highlight the benchmarks we can expect in the coming decades for 2D material CMOS technology for the 0.7 nm technology node.

CMOS technology is one of the greatest inventions of mankind and has been the most important driving force for almost every technology we have today. CMOS device downsizing based on silicon technology will end very soon, but it will be used for mainstream devices and integration technology for decades. However, new technology with enhanced performances and richer functionalities due to the incorporation of new materials, device structures, and functional modules, is on the horizon. There is still a long way to go, and so we hope the series of these Special Issues will draw attention from multidisciplinary experts addressing topical issues in their research and facilitate the exchange of views on the future of CMOS technology development. The second issue of “Abridging the CMOS Technology” is now open for submission and it will be published in 2024 by *Nanomaterials*.
